# The role of MEF2 transcription factors in dehydration and anoxia survival in *Rana sylvatica* skeletal muscle

**DOI:** 10.7717/peerj.4014

**Published:** 2017-11-09

**Authors:** Myriam P. Hoyeck, Hanane Hadj-Moussa, Kenneth B. Storey

**Affiliations:** Institute of Biochemistry, Departments of Biology and Chemistry, Carleton University, Ottawa, Canada

**Keywords:** Myocyte Enhancer Factor 2, Stress response, Hypometabolism, Wood frog, Pro-survival

## Abstract

The wood frog (*Rana sylvatica*) can endure freezing of up to 65% of total body water during winter. When frozen, wood frogs enter a dormant state characterized by a cessation of vital functions (i.e., no heartbeat, blood circulation, breathing, brain activity, or movement). Wood frogs utilize various behavioural and biochemical adaptations to survive extreme freezing and component anoxia and dehydration stresses, including a global suppression of metabolic functions and gene expression. The stress-responsive myocyte enhancer factor-2 (MEF2) transcription factor family regulates the selective expression of genes involved in glucose transport, protein quality control, and phosphagen homeostasis. This study examined the role of MEF2A and MEF2C proteins as well as select downstream targets (glucose transporter-4, calreticulin, and muscle and brain creatine kinase isozymes) in 40% dehydration and 24 h anoxia exposure at the transcriptional, translational, and post-translational levels using qRT-PCR, immunoblotting, and subcellular localization.* Mef2a/c* transcript levels remained constant during dehydration and anoxia. Total, cytoplasmic, and nuclear MEF2A/C and phospho-MEF2A/C protein levels remained constant during dehydration, whereas a decrease in total MEF2C levels was observed during rehydration. Total and phospho-MEF2A levels remained constant during anoxia, whereas total MEF2C levels decreased during 24 h anoxia and P-MEF2C levels increased during 4 h anoxia. In contrast, cytoplasmic MEF2A levels and nuclear phospho-MEF2A/C levels were upregulated during anoxia. MEF2 downstream targets remained constant during dehydration and anoxia, with the exception of *glut4* which was upregulated during anoxia. These results suggest that the upregulated MEF2 response reported in wood frogs during freezing may in part stem from their cellular responses to surviving prolonged anoxia, rather than dehydration, leading to an increase in GLUT4 expression which may have an important role during anoxia survival.

## Introduction

Winter in northern climates impose environmental challenges on native animals that are faced with limited water and food availability, reduced photoperiods, and sub-zero temperatures. As such, these animals have developed extreme overwintering survival strategies or have adapted to endure long migrations to warmer climates upon the onset of winter (Kiss et al., 2011; Storey & Storey, 2004a; Storey & Storey, 2017). A subset of animals have developed specialized energy-saving strategies that include freeze avoidance (circumvent freezing through the use of antifreeze-like molecules and supercooling mechanisms) and freeze tolerance (tolerate the accumulation of extracellular ice formation). Freeze tolerance is amongst the most extreme winter survival strategies and has been extensively studied in a variety of species, ranging from microorganisms, insects, amphibians (frogs and salamanders), and reptiles (turtles and snakes) (Storey & Storey, 2004a; Storey & Storey, 2005; Storey & Storey, 2017). The wood frog, *Rana sylvatica*, is the most northern frog in the world with a habitat that stretches across the boreal and eastern deciduous forests of North America (Conlon et al., 1998; Costanzo et al., 2015; Storey & Storey, 2017). Consequently, the wood frog displays amongst the highest capacity for freeze tolerance, enduring freezing of up to 65% of total body water as extracellular ice (Layne & Lee, 1986; Storey & Storey, 1996). Freezing is initiated by the slow and controlled nucleation of extracellular ice at temperatures just below the freezing point of water (−0.5 °C), providing sufficient time for the frog to induce the physiological and biochemical reorganization required for freeze survival. These adaptations include the synthesis of cryoprotectant glucose and the suppression of metabolic rate (Costanzo & Lee Jr, 1993; Storey, 2004; Storey & Storey, 2004c).

Winter hardiness strategies are associated with a myriad of physiological and molecular strategies. When frozen, the wood frog enters a state of suspended animation characterized by a cessation of vital functions such as a lack of heart beat, blood circulation, breathing, brain activity, and movement (Layne, Lee & Heil, 1989; Storey & Storey, 1996). This cessation of vital functions during freezing imposes cellular stress on the cells including dehydration, anoxia, ischemia, and osmotic stress as the transport of oxygen and fuels to the cells is impaired and water is sequestered as ice into extracellular space. Freezing survival by the wood frog is aided by a natural extreme tolerance for whole body dehydration; the frog can endure the loss of 65–70% of total body water, leading to significant changes in cell volume and osmolality (Churchill & Storey, 1993). To prevent damage to the plasma membrane and cytoskeleton, the wood frog accumulates large quantities of osmolytes. Indeed, to reduce freezing injury, the wood frog relies on the colligative properties of glucose, accumulating up to 200–300 mM of glucose by activating liver glycogenolysis within minutes of the initial ice nucleation event (Storey, 2004). Furthermore, as vital functions come to a halt, cells become anoxic which inhibits aerobic metabolism via the TCA (tricarboxylic acid) cycle and ETC (electron transport chain) leading to a decrease in ATP production as the frog relies solely on anaerobic glycolysis (Storey & Storey, 2017). Global metabolic rate depression (MRD) is a core component of the wood frog’s freeze tolerance response which reduces organismal energy demands by suppressing energy expensive process (i.e., transcription, translation, reproduction, cell cycle, and ATP-dependent ion pumps), allowing existing ATP pools to be reprioritized to cellular functions that are vital for survival (i.e., enhance cellular defense strategies such as: antioxidants, chaperone proteins, anti-apoptosis, antimicrobial, and protection from mechanical ice damage) (Biggar & Storey, 2011; Dawson, 2014; Gerber, Wijenayake & Storey, 2016; Storey, 2015; Storey & Storey, 2017). Transitions into hypometabolic states generally lowers metabolic rates to as low as 1–30% of the normal resting rates (Storey & Storey, 2017). Hypometabolism is controlled through diverse biochemical processes ranging from reversible protein phosphorylation, histone post-translational modifications, supressed gene transcription and protein translation, and altered microRNA expression (Storey & Storey, 2017).

Many pro-survival pathways that help facilitate the smooth transition between freeze-thaw cycles have also been implicated in other adaptational responses to environmental stress (Storey, 2004). Current research has indicated a role of the Myocyte Enhancer Factor-2 (MEF2) family of transcription factors in overwintering stress responses (Aguilar, Hadj-Moussa & Storey, 2017; Tessier & Storey, 2010; Tessier & Storey, 2012). Initially, MEF2 proteins where identified in skeletal muscle and were only thought to regulate genes involved in muscle functioning. However, MEF2 factors have now been shown to be ubiquitously expressed and play various roles in cell differentiation, proliferation, morphogenesis, survival, and apoptosis (Perry et al., 2009; Pon & Marra, 2016; Potthoff & Olson, 2007; Ramachandran et al., 2008; Wales et al., 2014). In vertebrates, MEF2 transcription factors are encoded by four genes- *mef2a*, *b*, *c*, and *d* which display a tissue-specific and temporal-dependent expression pattern (Gregoire et al., 2006; Perry et al., 2009; Potthoff & Olson, 2007; Wales et al., 2014). MEF2 proteins are members of the MADS-box super family of transcriptional regulatory proteins. MEF2 factors contain a MADS-box and a MEF2 domain at their N-terminal involved in dimerization, DNA binding, and co-factor interaction, whereas the C-terminal domain contains a bipartite nuclear localization signal (NLS) required for subcellular localization and transcriptional activation (Borghi et al., 2001; Desjardins & Naya, 2016; Perry et al., 2009; Potthoff & Olson, 2007). In the basal state, DNA-bound MEF2 proteins remain inactive through association with class II histone deacetylases (HDACs), and other corepressors such as Cabin 1 and MITR, via the MADS domain. Extracellular signals will induce signaling cascades that will activate MEF2 via post-translational modifications (PTMs), with phosphorylation being the main PTM regulating MEF2 activity. Phosphorylation of conserved serine residues on MEF2-associated HDAC proteins by calcium-dependent kinases (i.e., CaMKs and PKDs) relieves HDAC-induced repression and stimulate HDAC export to the cytoplasm. Direct phosphorylation of the MEF2 transcriptional activation domain at various threonine residues by MAP (mitogen-activated protein) kinases will enhance MEF2 activity, triggering MEF2-dependent gene expression (Borghi et al., 2001; Gregoire et al., 2007; Mcgee & Hargreaves, 2004; Potthoff & Olson, 2007).

During freezing the wood frog remains immobile for extended periods of time which may lead to a loss in muscle mass and strength, rendering the muscle vulnerable to disuse atrophy. Consequently, the wood frog requires specialized adaptations to preserve muscle structure and function by altering metabolic capacity and muscle fibre composition (Costanzo et al., 2013; Costanzo et al., 2015). The MEF2 factors regulate gene expression of proteins that play essential roles in muscle function and facilitating freeze tolerance, as well as pro-survival responses, including the glucose transporter 4 (GLUT4), calreticulin (CALR), and creatine kinase isozymes (CKM and CKB). GLUT4 is an insulin-dependent glucose transporter that is primarily found in skeletal muscle and is required for the maintenance of glucose homeostasis (Olson, 2012). GLUT4 also plays a crucial role in the uptake of glucose as a cryoprotectant in wood frog muscles as the frog transitions into a frozen state (Storey, 2004). Calreticulin (Calr) resides in the endoplasmic reticulum lumen where it maintains extracellular Ca^2 +^ homeostasis and functions as a chaperone protein responsible for protein folding and quality control (Lu, Weng & Lee, 2015; Michalak et al., 2009). Chaperone proteins play an integral role in freeze tolerance to maintain functional proteins under stress (Storey & Storey, 2017). Muscle and brain creatine kinase (CK) isozymes are expressed in skeletal muscle and have a central role in the phosphocreatine circuit involved in the synthesis and breakdown of phosphocreatine through the exchange of high-energy phosphates with ATP. Phosphocreatine functions as an energy store used to replenish ATP when ATP production is reduced under stress (Deursen et al., 1994; Storey & Storey, 2017).

Recent research has shown an upregulation in MEF2 transcript and protein expression, as well as an increase in *glut4*, *calr*, *ckm*, and *ckb* transcript levels in wood frog skeletal muscle during 24 h freezing (Aguilar, Hadj-Moussa & Storey, 2017). This research suggests that MEF2 transcription factors play an important role in freeze-tolerance. As such, the present study aimed to further dissect the MEF2 response in wood frog skeletal muscle during dehydration and anoxia to determine whether cellular responses seen in the wood frog during freezing are induced by associated component stresses. Immunoblotting, qRT-PCR, and subcellular localization were used to examine the stress-induced transcriptional, translation, and post-translational activation of MEF2 transcription factors (MEF2A and MEF2C) and selected downstream targets (*glut4*, *calreticulin*, *ckm*, and *ckb*) in wood frog skeletal muscle comparing control, 40% dehydration, rehydration, 4 h anoxia, and 24 h anoxia exposures. Overall, MEF2 activity increased during anoxia which correlated with an increase in *glut4* expression, while no changes were observed during dehydration. These results indicate a possible role of the GLUT4 transporter during anoxia survival and that the wood frog’s MEF2 response during freezing may in part stem from cellular responses to prolonged anoxia rather than dehydration.

## Materials and Methods

### Animals

Mature male wood frogs were captured from spring breeding ponds near Oxford Mills, Ontario, Canada in mid-April. Frogs were washed in a tetracycline bath and placed in plastic containers with damp sphagnum moss at 5 °C for 1–2 weeks prior to experimentation. Control frogs were randomly sampled under this condition. For dehydration experiments, frogs were weighed and placed in desiccation jars at 5 °C. The frogs were weighed at 12 h intervals until 40% dehydration of total body water was attained, after which half the frogs (chosen randomly) were sampled while the other half were placed in a container with 0.5 cm of distilled water for rehydration. The percentage of total body water lost was calculated from the change in mass of the frogs over time, using the following equation:

\begin{eqnarray*}\text{% water loss}= \frac{ \left( {M}_{i}-{M}_{d} \right) }{ \left( {M}_{i}\times BW{C}_{i} \right) } \times 100\text{%} \end{eqnarray*}

where *M*_*i*_ is the initial mass of the frog, *M*_*d*_ is the mass at each weighing during the dehydration procedure, and *BWC*_*i*_ is the initial body water content of the frog prior to dehydration (note: for control frog *BWC*_*i*_ = 80.8 ± 1.2%). Three experimental conditions were tested: control, 40% dehydration, and full rehydration with *n* = 4 per condition. Frogs were subjected to 40% dehydration to mimic the cellular dehydration experienced during freezing, allowing biochemical adaptations required to survive prolonged dehydration during freezing to be analyzed (Storey & Storey, 2001).

For anoxia, the experimental containers consisted of plastic jars with lids that had two syringe ports which allowed flushing with nitrogen gas. Plastic jars were lined with damp paper towels (wetted with distilled water that had been bubbled with 100% nitrogen gas), chilled on ice and then flushed with nitrogen gas for 20 min. Frogs were then placed in the jars (5–6 per jar), the lids were tightened, and the jars were again flushed with nitrogen gas for 30 min, after which the ports were closed and the jars were sealed with Parafilm. Frogs were incubated at 5 °C for 4 h and 24 h. Following anoxia exposure, jars were placed on ice and flushed with N_2_, from which the frogs were quickly sampled. Three experimental conditions were tested: control, 4 h, and 24 h anoxia with *n* = 4 per condition. Frogs were subjected to 24 h anoxic conditions to induce maximal chronic anoxia and trigger physiological/biochemical changes that are required and maintained throughout the winter, whereas 4 h anoxia generated acute anoxic conditions experienced during early transitions into the frozen state (Lee et al., 1992; Storey & Storey, 2017). Frogs under all conditions described above were euthanized by pithing and skeletal muscle tissue was quickly excised and flash frozen using liquid N_2_. Samples were stored at −80 °C until use.

The Carleton University Animal Care Committee, in accordance with the Canadian Council on Animal Care guidelines, approved all animal handling protocols and experiments performed in this study (Protocol #13683). Wildlife Scientific Collector’s Authorization was granted by the Ministry of Natural Resources, Ontario authorization #1085726.

### Protein extraction

Total soluble protein extracts were prepared from wood frog skeletal muscle under control, 40% dehydration, full rehydration, 4 h anoxia, and 24 h anoxia conditions. Approximately 0.5 g of tissue were powdered and homogenized with a Polytron PT10 homogenizer in 1 mL of homogenizing buffer (20 mM HEPES, 200 mM NaCl, 0.1 mM EDTA, 10 mM NaF, 1 mM Na_3_VO_4_, 10 mM β-glycerophosphate, 10 µM PMSF, pH 7.5) with 10 µL/mL Sigma protease inhibitor cocktail (Bioshop, Catalog #PIC001: 104 mM AEBSF, 80 µM aprotinin, 4 mM bestatin, 1.4 mM E-64, 2 mM leupeptin, 1.5 mM pepstatin A). Samples were centrifuged for 15 min at 10,000 rpm at 4 °C and the supernatant was collected. Nuclear and cytoplasmic extracts were prepared as previously described with a few alterations (Aguilar, Hadj-Moussa & Storey, 2016). In brief, skeletal muscle of control, 40% dehydrated, and 24 h anoxic wood frogs was homogenized in buffer (10 mM HEPES, 10 mM KCl, 10 mM EDTA, 1 mM DTT, 1 mM PMSF, pH 7.9) using a Dounce homogenizer. Lysates were centrifuged for 10 min at 8,000 g and at 4 °C, and the supernatant was collected as the cytoplasmic fractions. Pellets were resuspended in 150 µL of nuclear extraction buffer (10 mM HEPES, 400 mM NaCl, 1 mM EDTA, 1 mM DTT, 10% v:v glycerol, 1.5 µM PMSF, pH 7.9) and slowly mixed for 1 h at 4 °C. Samples were centrifuged for 10 min at 10,000 rpm and 4 °C, and the supernatant was collected as the nuclear fractions. The soluble protein concentrations of total, cytoplasmic, and nuclear extracts were quantified using the BioRad protein assay (Biorad, Catalog #500-0006). Samples were adjusted to a concentration of 10 µg/µL with homogenization buffer. Aliquots were then combined 1:1 v:v with 2X SDS loading buffer (100 mM Tris-base [pH 6.8], 4% w:v SDS, 20% v:v glycerol, 0.2% w:v bromophenol blue, 10% v:v 2-mercaptoethanol) to a final concentration of 5 µg/µL (control) and 2.5 µg/µL (stress). Samples were then boiled for 10 min and stored at −40 °C for further use.

### Immunoblotting

Aliquots containing 20 µg of protein, 5 µL of pre-stained protein molecular weight ladder (Catalog #PM005-0500; FroggaBio, Toronto, ON, Canada), and 4 µL of mammalian positive control were loaded onto 10% SDS polyacrylamide gels. Proteins were resolved by electrophoresis at 180 V for 75–120 min in 1× Tris-glycine running buffer (0.25 M Tris-base, 2.45 M glycine, and 0.035 M SDS) using a BioRad Mini Protean III system. Proteins were electroblotted by wet transfer onto a 0.45 µm polyvinylidene difluoride membrane (Catalog #IPVH00010; Millipore, Burlington, MA, USA) in 1× transfer buffer (25 mM Tris, 192 mM glycine, and 10% v:v methanol, pH 8.5) at 160 mA for 90 min using a BioRad Mini-Protean Transfer cell. Membranes were washed in 0.5× TBST (20 mM Tris-base, 140 mM NaCl, 0.05% v:v Tween-20, pH 7.5) and then blocked using 5–10% w:v milk for 30 min–1 h or 2 mg/mL polyvinyl alcohol (PVA; 70–100 kDa) for 30 sec–1 min, depending on the protein target. The membranes were then washed with 0.5× TBST and probed with 5 mL of antigen-specific primary antibody (1:1,000) at 4 °C for 24 h on a rocker. The antibodies used in this experiment were purchased from Santa Cruz Biotechnology (MEF2A and phospho-MEF2A^Thr312^; Santa Cruz Biotechnology, Santa Cruz, CA, USA), and GenScript (MEF2C and phospho-MEF2C^Thr300^; GenScript, Nanjing, China). As the wood frog is not genome-sequenced, multi-sequence protein alignments were preformed to confirm that the epitopes targeted by the antibodies used in this study displayed a high-degree of specificity and conservation across a diverse range of species including the frog *Xenopus laevis*.

Following incubation with the primary antibody, membranes were washed with 0.5× TBST and incubated for 40 min at room temperature with hP-conjugated anti-rabbit IgG secondary antibody (Catalog #APA007P; Bioshop, Burlington, ON, Canada) diluted 1:8,000 v:v in 0.5× TBST. Membranes were then washed with 0.5× TBST and visualized with enhanced chemiluminescence solution (Millipore, Burlington, MA, USA) using a Chemi-Genius Bio-Imaging System (Syngene, Frederick, MD, USA). Membranes were stained with Coomassie blue (0.25% w:v Coomassie brilliant blue, 7.5% v:v acetic acid, and 50% methanol) to visualize the proteins.

### RNA isolation

Total RNA was extracted from the skeletal muscle of control, 40% dehydrated, and 24 h anoxic wood frogs. Approximately 50–100 mg samples of frozen tissue were powdered and homogenized in 1 mL Trizol reagent (Cat #15596-018; Invitrogen, Carlsbad, CA, USA) using a Polytron PT1200 homogenizer (Kinematica, Luzern, Switzerland). 200 µL of chloroform were added and samples were centrifuged for 15 min at 12,000 rpm and 4 °C. The supernatant was transferred to microcentrifuge tubes and samples were mixed with 500 µL of isopropanol. Samples were left on ice for 10 min to allow RNA precipitation. Samples were centrifuged for 15 min at 12,000 rpm at room temperature. The supernatant was discarded and the pellets were washed with 1 mL of 70% ethanol, after which the sample were centrifuged for 5 min at 7,500 rpm at room temperature, and the supernatant was aspirated. Pellets were air-dried for ∼10 min and then resuspended in 30–50 µL RNase-free water.

RNA concentration and purity was determined by the 260/280 ratio measured using a Take3 micro-volume quantification plate (BioTek, Winooski, VT, USA) and a PowerWave HT spectrophotometer (BioTek, Winooski, VT, USA). Samples with a 260/280 ratio of ∼2.0 were used for transcript analysis. RNA integrity was assessed by verifying the presence of sharp 28S and 18S ribosomal RNA bands on a 1% agarose gel electrophoresis stained with SYBR Green (Cat #S7563; Invitrogen, Carlsbad, CA, USA). Samples were frozen at −20 °C until use.

### cDNA synthesis

First strand synthesis was performed using 3 µg of total RNA diluted in DEPC-treated H_2_O to obtain a final volume of 10 µL. 1 µL of 200 ng/µL oligo(dT) (5′-TTTTTTTTTTTTTTTTTTTTTV-3′; V = A or G or C; Sigma Genosys) was added to the samples, and samples were incubated in a thermocycler (Mastercycler Eppendorf) at 65 °C for 5 min, after which they were chilled on ice for 1 min. Samples were then incubated at 42 °C for 45-60 min in an Eppendorf thermocycler (Mississauga, ON, Canada) with 4 µL of 5× first strand buffer (Invitrogen), 2 µL of 0.1 M DTT (Invitrogen, Carlsbad, CA, USA), 1 µL of 10 mM dNTPs (BioShop, Burlington, ON, Canada), and 1 µL MMLV Reverse Transcriptase (Invitrogen, Carlsbad, CA, USA). Serial dilutions of the cDNA were prepared and stored at 4 °C until use.

### qRT-PCR amplification

Primer sequences used in this study were from Aguilar, Hadj-Moussa & Storey (2017). qRT-PCR assays were performed as described by Pellissier et al. (2006) using a Bio-Rad MyiQ_2_ Detection System (BioRad, Hercules, CA, USA) following MIQE guidelines (Bustin et al., 2009). 20 µL reactions were used, each consisting of: 2 µL cDNA, 2 µL qRT-PCR buffer (100 mM Tris–HCl [pH 8.5], 500 mM KCl, 1.5% Triton X-100, 20 mM MgCl_2_, 2 mM dNTPs, and 100 nM fluorescein), 0.16 µL of 25 mM dNTPs, 4 µL of 1 M trehalose (Cat #TRE222; BioShop, Burlington, ON, Canada), 0.5 µL of 100% formamide (BioShop, Cat #FOR001), 0.1 µL of 100× SYBR Green diluted in DMSO (Cat #S7585; Invitrogen, Carlsbad, CA, USA), 0.5 µL of 0.3 nmol/µL forward primer, 0.5 µL of 0.3 nmol/µL reverse primer, 0.125 µL of 5U/µL Taq Polymerase (Cat #TAQ001.1; BioShop, Burlington, ON, Canada), and 10.115 µL DEPC-treated water. The optimized PCR protocol consisted of an initial denaturing step at 95 °C for 2 min, followed by 60 cycles of: 95 °C for 45 sec, the primer-specific optimal annealing temperature for 45 sec, and 72 °C for 45 sec; and a final step of 72 °C for 4 min. The following annealing temperatures were used: 59.5 °C for *mef2a* and *mef2c*, 53 °C for *ckm* and *ckb*, and 52 °C for *calr*, *glut4*, and *α*-*tubulin*. Primer efficiency was verified using a post-run melt-curve analysis. Reactions that amplified multiple products were not used. PCR amplicons were gel purified using a 1% agarose gel stained with SYBR Green, sequenced at the Ontario Health Research Institute (OHRI, Ottawa, ON), and validate using BLAST (http://blast.ncbi.nlm.nih.gov/).

### Data analysis and statistics

Target protein bands were identified using a molecular weight ladder (Cat #PM005-0500; FroggaBio, Toronto, ON, Canada) and a mammalian positive control (thirteen-lined ground squirrel). Immunoblot and Coomassie-stained protein band densities were quantified using Gene Tools software. Immunoblot bands were normalized against the summed intensity of a group of Coomassie-stained protein bands in the same lane that exhibited constant expression amongst the three experimental conditions, without including the band of interest.

*α*-tubulin was used as a reference gene because it exhibited stable expression levels in *R. sylvatica* skeletal muscle under control, dehydration, and anoxic conditions. Amplification curves of each PCR amplicon were normalized against *α*-tubulin transcript levels of each cDNA sample using the Pfaffl method. A one-way ANOVA was performed and significant differences between control and stress groups was further analyzed using a post-hoc Dunnett’s test. Data was expressed as means ± SEM, *n* = 3–4 independent biological replicates from different animals, *p*-value threshold for significance was *p* < 0.05. Statistical analyses and figure generation were conduced using 3.3.2 RBioplot software (Zhang & Storey, 2016).

## Results

### Analysis of mef2 transcript levels in response to dehydration and anoxia

**Figure 1:**
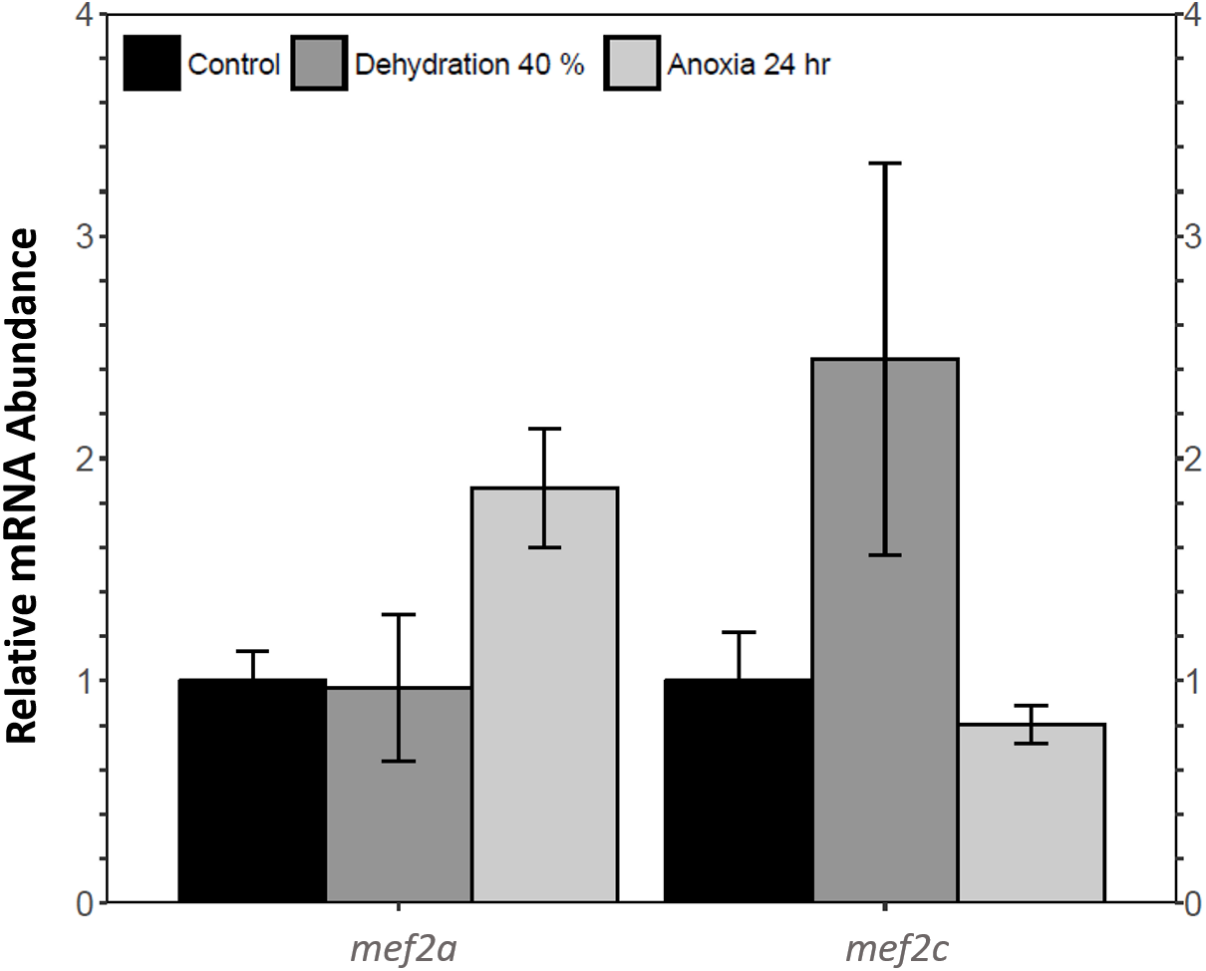
Analysis of *mef2* genes in the wood frog skeletal muscle under control, 40% dehydration, and 24 h anoxia conditions, as determined using qRT-PCR. Histogram displays standardized mRNA abundance of *mef2a* and *mef2c* relative to control. Transcript levels were normalized using *α*-*tubulin* as the internal control. Data are means ± SEM, *n* = 3–4 independent biological replicates. Statistical analysis was preformed using a one-way ANOVA with a Dunnett’s post-hoc test. * significantly different from control (*p* < 0.05).

Relative *mef2a* and *mef2c* mRNA abundance in wood frog skeletal muscle during 40% dehydration and 24 h anoxia was analysed using qRT-PCR. *Mef2a* and *mef2c* transcript levels remained constant during both 40% dehydration and 24 h anoxia (Fig. 1).

### MEF2 protein abundance during dehydration, rehydration, and anoxia

**Figure 2:**
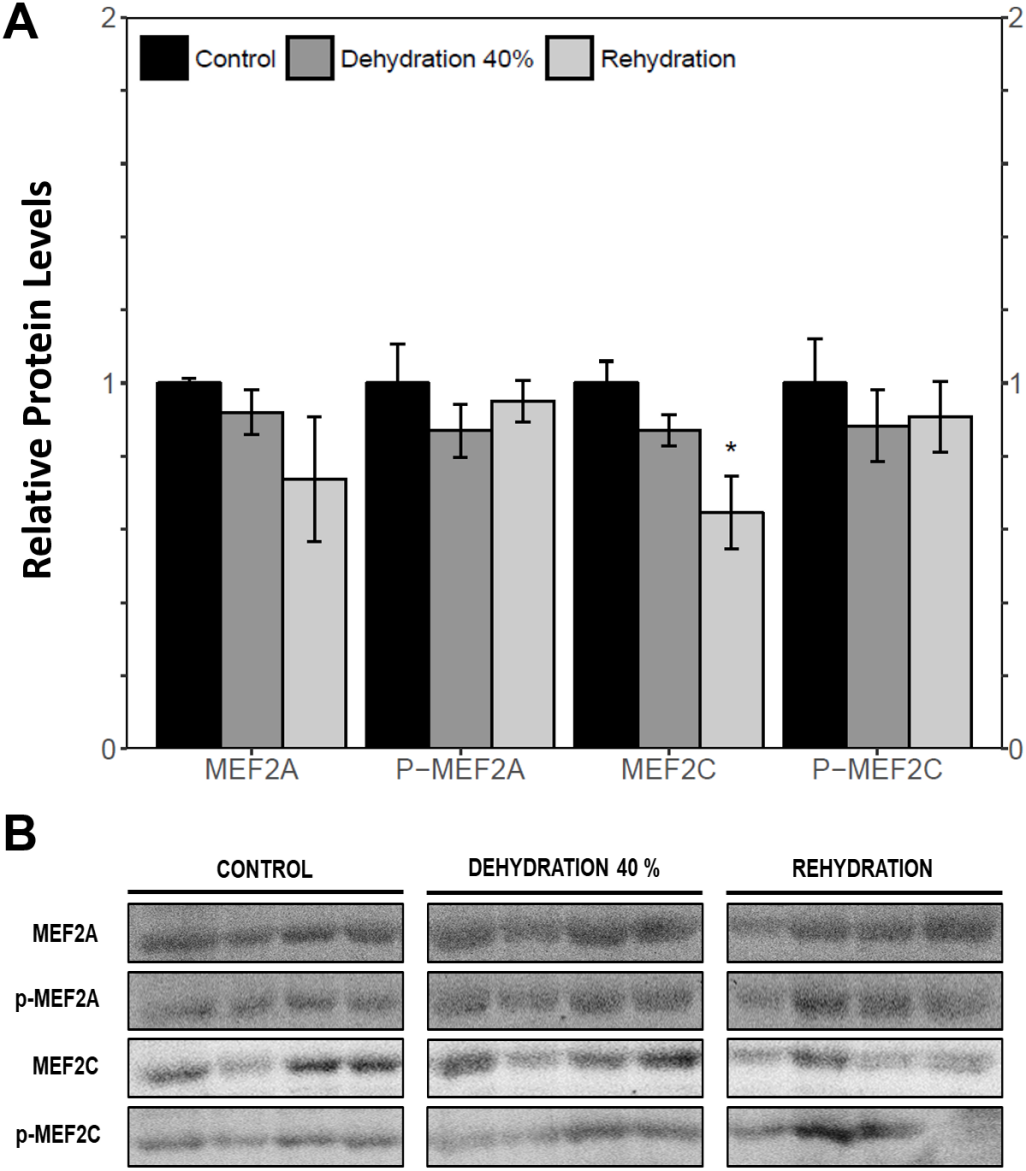
Relative protein abundance of total and phosphorylated forms of MEF2A/C in wood frog skeletal muscle under control, 40% dehydration, and recovery conditions, as determined by immunoblotting. (A) displays the histogram of relative protein abundance. (B) shows representative immunoblots from each group. Data are means ± SEM, *n* = 3–4 independent biological replicates. Statistical analysis was performed using a one-way ANOVA with a Dunnett’s post-hoc test, *significantly different from control (*p* < 0.05).

**Figure 3:**
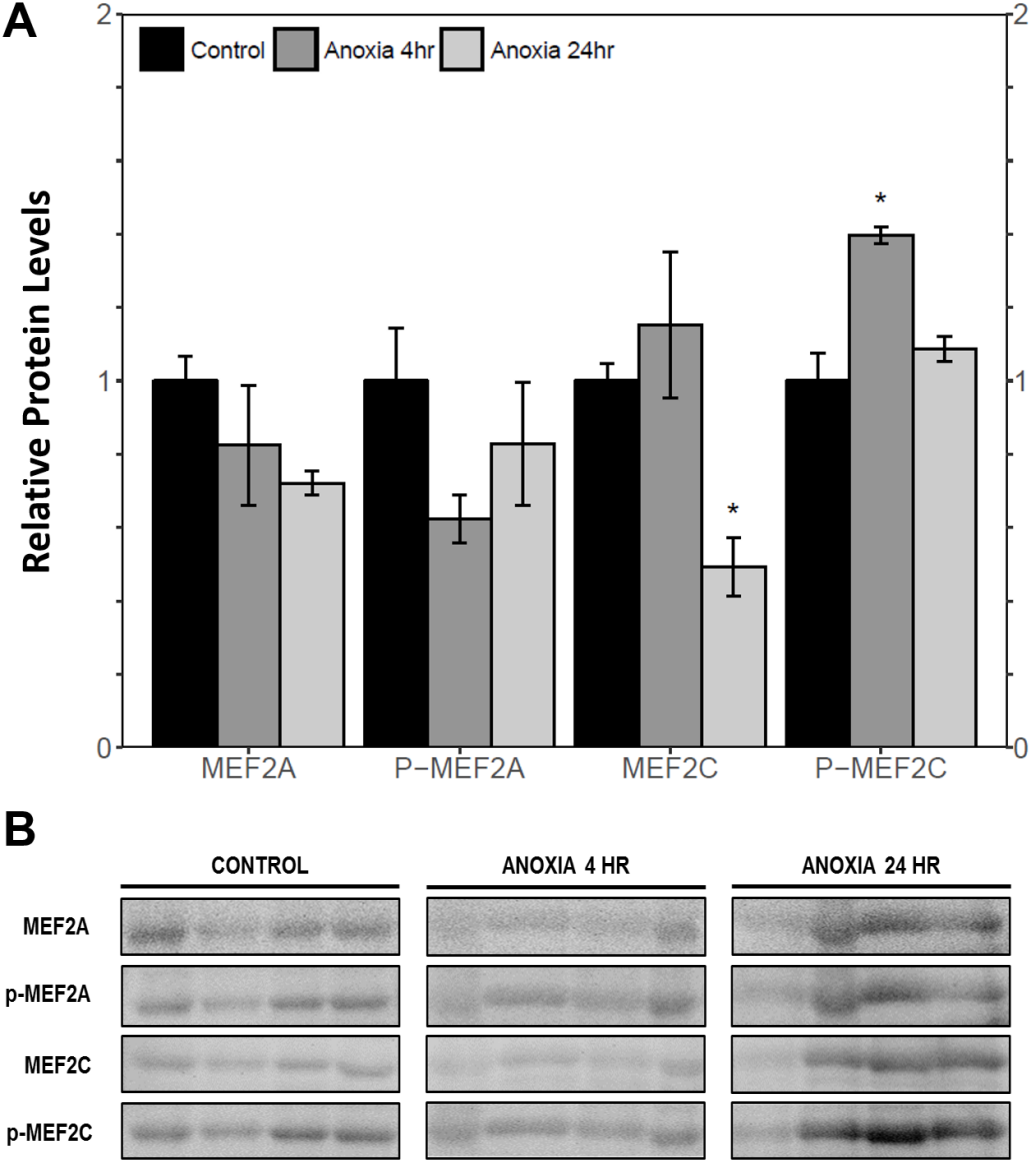
Relative protein abundance of total and phosphorylated forms of MEF2A/C in wood frog skeletal muscle under control, 4 h anoxia, and 24 h anoxia, as determined by immunoblotting. (A) displays the histogram of relative protein abundance. (B) shows representative immunoblots from each group. Data are means ± SEM, *n* = 3–4 independent biological replicates. Statistical analysis was performed using a one-way ANOVA with a Dunnett’s post-hoc test, *significantly different from control (*p* < 0.05).

Immunoblotting was used to analyse the relative protein levels of total and phosphorylated MEF2A/C in wood frog skeletal muscle under control, 40% dehydration, rehydration, 4 h anoxia, and 24 h anoxia conditions. During 40% dehydration, total and phosphorylated MEF2A/C were found to remain constant in comparison to control conditions (Fig. 2). Likewise, no change in MEF2 protein levels was observed during rehydration with the exception of total MEF2C which showed a 35.4 ± 10.0% decrease (*p* < 0.05). During 4 h anoxia, total MEF2A/C and phospho-MEF2A^Thr312^ remained constant, whereas phospho-MEF2C^Thr300^ showed a 1.40 ± 0.024 fold increase (*p* < 0.05) (Fig. 3). Total and phospho-MEF2A^Thr312^ and phospho-MEF2C^Thr300^ remained constant during 24 h anoxia, while total MEF2C showed a 50.7 ± 7.5% decrease (*p* < 0.05).

### MEF2 subcellular localization in response to dehydration and anoxia

**Figure 4:**
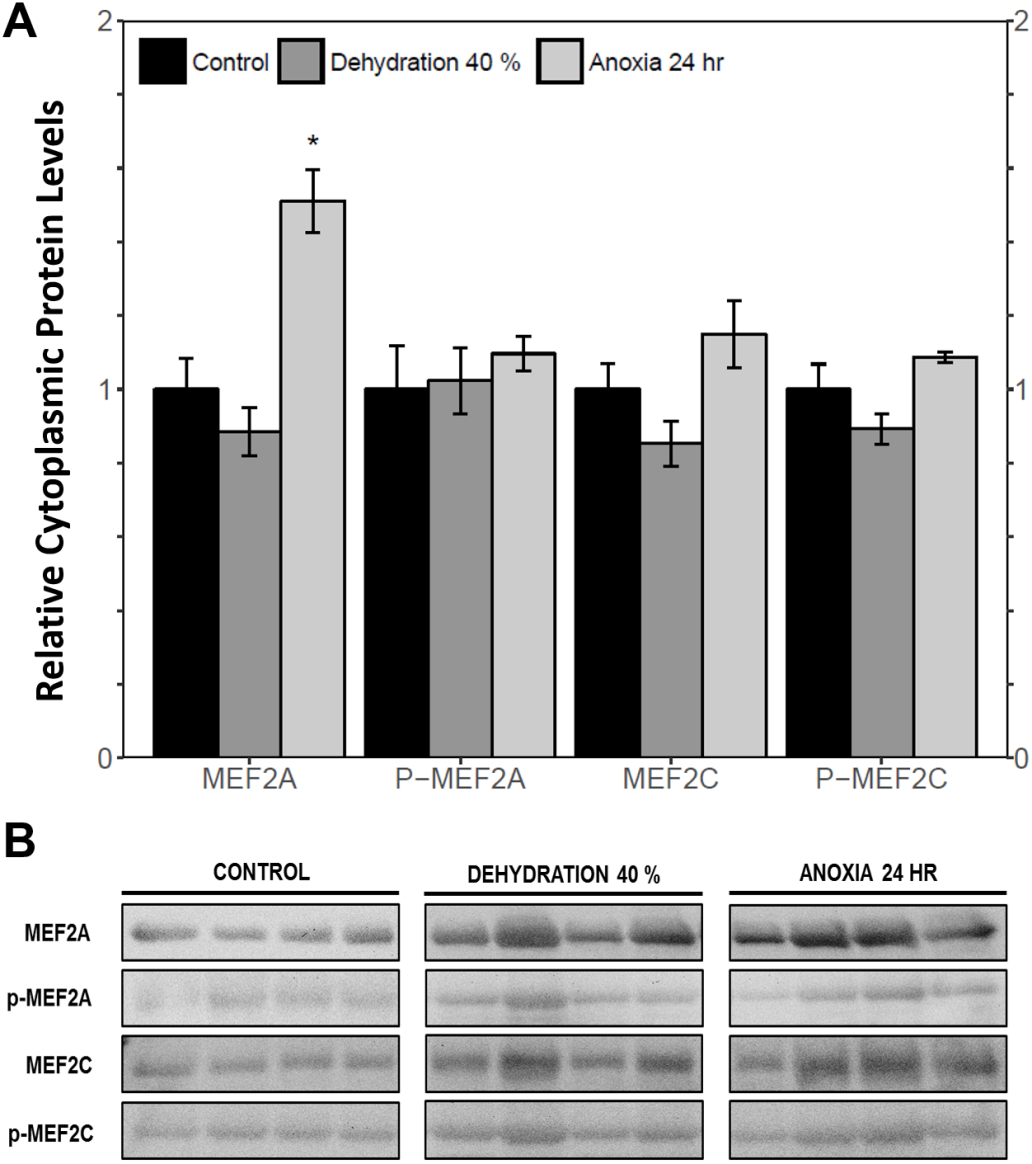
Cytoplasmic distribution of total and phosphorylated forms of MEF2 transcrition factors in wood frog skeletal muscle under control, 40% dehydration, and 24 h anoxia, as determined by immunoblotting. (A) displays the histogram of relative cytoplasmic protein levels of total MEF2A/C and phosphorylated MEF2A^Thr312^ and MEF2C^Thr300^ relative to control conditions. (B) shows representative immunoblots from each group. Data are means ± SEM, *n* = 3–4 independent biological replicates. Statistical analysis was performed using a one-way ANOVA with a Dunnett’s post-hoc test, *significantly different from the control (*p* < 0.05).

**Figure 5:**
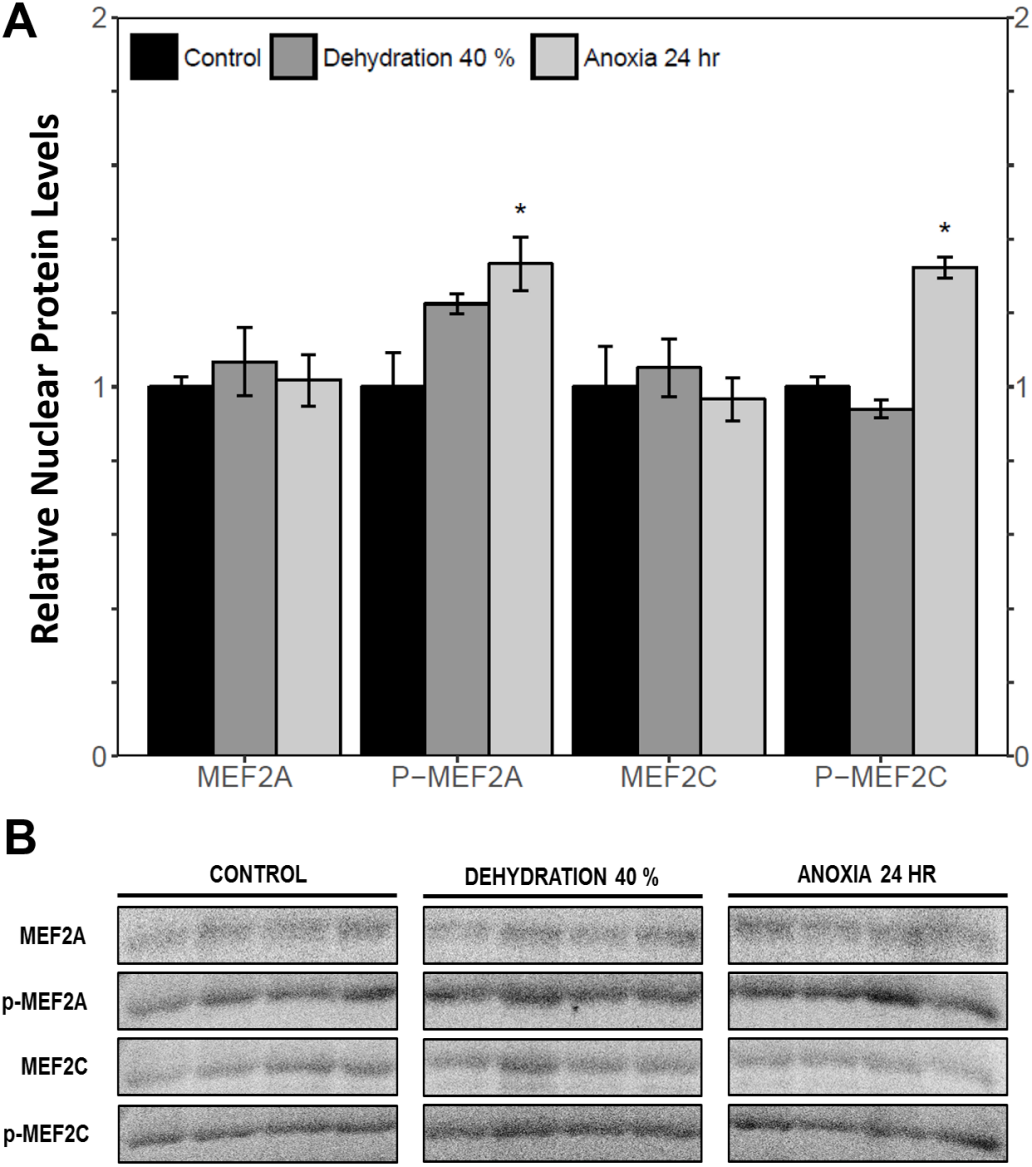
Nuclear distribution of total and phosphorylated forms of MEF2 proteins in wood frog skeletal muscle under control, 40% dehydration, and 24 h anoxia, as determined using immunoblotting. (A) displays the histogram of relative nuclear protein levels of total MEF2A/C and phosphorylated MEF2A^Thr312^ and MEF2C^Thr300^ relative to control conditions. (B) shows representative immunoblots from each group. Data are means ± SEM, *n* = 3–4 independent biological replicates. Statistical analysis was performed using a one-way ANOVA with a Dunnett’s post-hoc test, *significantly different from the control (*p* < 0.05).

The cytoplasmic and nuclear subcellular localization of total and phosphorylated MEF2A/C in the wood frog skeletal muscle under control, 40% dehydration, and 24 h anoxia conditions were analysed using immunoblotting. It was observed that during 40% dehydration, the cytoplasmic and nuclear distribution of total and phosphorylated MEF2A/C remained constant relative to control conditions (Figs. 4 and 5). During 24 h anoxia, relative cytoplasmic protein levels of total MEF2A increased by 1.51 ± 0.085 fold, whereas phospho-MEF2A^Thr312^, total MEF2C, and phospho-MEF2C^Thr300^ remained constant (Fig. 4). In contrast, relative nuclear protein levels of phospho-MEF2A^Thr312^ and phospho-MEF2C^Thr300^ increased by 1.33 ± 0.071 and 1.32  ± 0.028 fold, respectively, whereas total MEF2A/C remained constant (Fig. 5).

### Transcriptional activation of MEF2 downstream target genes during dehydration and anoxia

**Figure 6:**
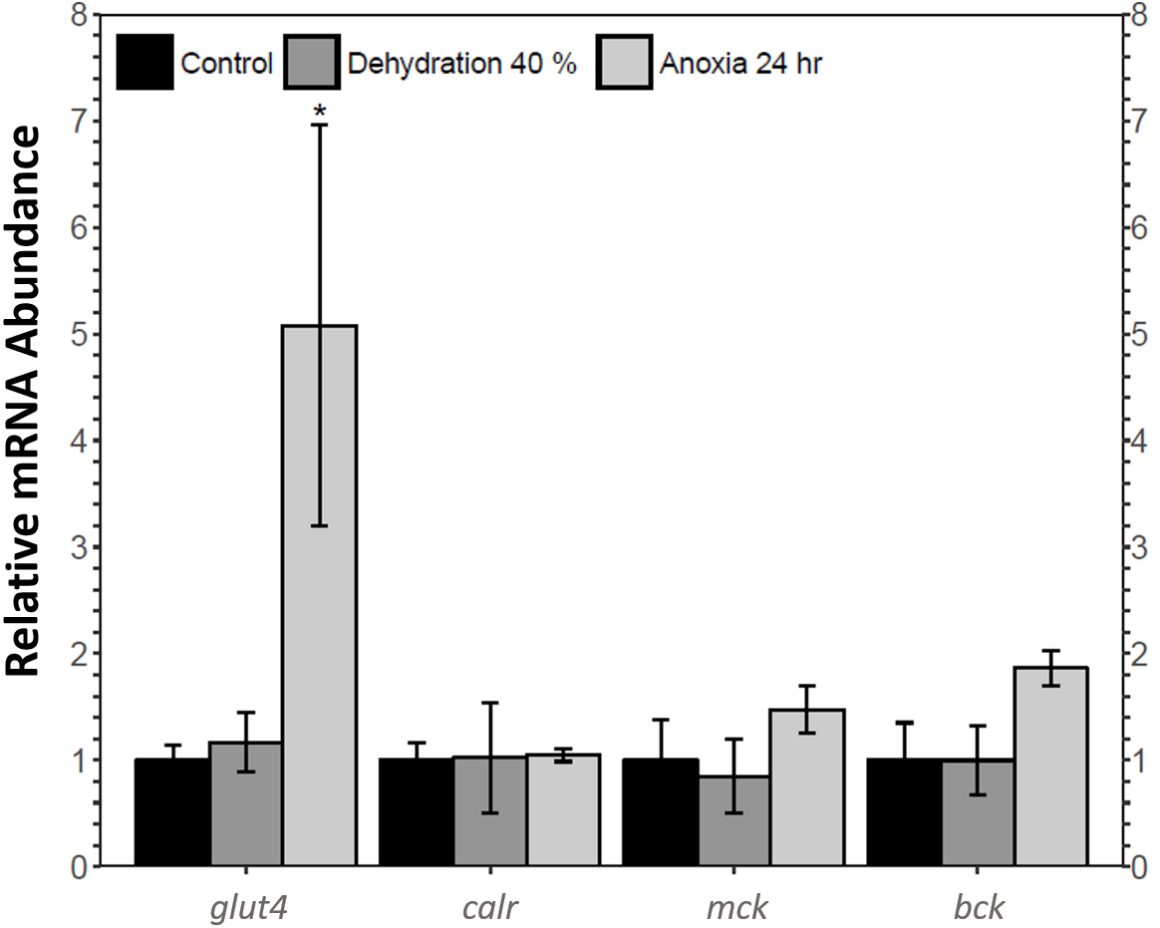
Analysis of *mef2* downstream gene targets in wood frog skeletal muscle under control, 40% dehydration, and 24 h anoxia conditions, as determined using qRT-PCR. Histogram displays mRNA abundance of *glut4*, *calr*, *ckm*, and *ckb* relative to control. Transcript levels were standardized using *α*-*tubulin* as an internal control. Data are means ± SEM, *n* = 3–4 independent biological replicates. Statistical analysis was performed using a one-way ANOVA with a Dunnett’s post-hoc test, *significantly different from the control (*p* < 0.05).

The relative mRNA levels of four MEF2 downstream target genes in the skeletal muscle of the wood frog during control, 40% dehydration, and 24 h anoxia were analysed using qRT-PCR: glucose transporter 4 (*glut4*), calreticulin (*calr*), muscle creatine kinase (*ckm*), and brain creatine kinase (*ckb*). During 40% dehydration, *glut4*, *calr*, *ckm*, and *ckb* transcript levels remained constant (Fig. 6). Likewise, *calr*, *ckm*, and *ckb* transcript levels remained constant during 24 h anoxia, whereas *glut4* increased by 5.08 ± 1.9 fold (*p* < 0.05).

## Discussion

Freeze tolerance is an ecologically relevant strategy used by the wood frog to survive subzero temperatures during the winter. As the frog freezes, the formation of extracellular ice imposes various cellular stresses including dehydration and anoxia. To survive freezing and its component stresses, the frog enters a state of suspended animation characterized by a global decrease in metabolic rate (Layne & Lee, 1986; Storey & Storey, 1996; Storey & Storey, 2017). Consequently, the frog remains inactive for extended periods of time putting it at risk of muscle atrophy. The wood frog has adapted various strategies to preserve muscle mass and mechanical properties allowing for a safe transition into the frozen and thawed states (Costanzo & Lee Jr, 1993; Costanzo et al., 2013; Costanzo et al., 2015). MEF2 proteins are key transcriptional regulators that are required for proper muscle function but also play a role in basic pro-survival processes such as glucose uptake, calcium homeostasis, protein quality control, and phosphocreatine synthesis/breakdown (Anderson et al., 2015; Mcgee & Hargreaves, 2004; Potthoff & Olson, 2007). The present study analysed the transcriptional, translational, and post-translational regulation of total and phosphorylated MEF2A and MEF2C in wood frog skeletal muscle under dehydration and anoxia stress. In addition, transcript expression of four MEF2 downstream target genes: glucose transporter-4, calreticulin, and muscle and brain creatine kinase isozymes, were examined to evaluate their potential function in dehydration and anoxia stress survival. This research showed that MEF2 proteins were anoxia-responsive leading to an increase in GLUT4 expression, whereas MEF2 activity remained unchanged during dehydration. This suggests that the wood frog’s MEF2 response is not only freeze-responsive but may also be triggered by prolonged anoxia rather than dehydration.

Past research has shown that MEF2-mediated gene transcription plays a significant role in muscle remodelling and adaptation during torpor-arousal cycles in the thirteen-lined ground squirrel (Tessier & Storey, 2010; Tessier & Storey, 2012). This suggested that MEF2 proteins may also play a role in protection against muscle atrophy in other organisms that transition into a hypometabolic state during stress. Indeed, a recent study focused on the role of MEF2 proteins in skeletal muscle of frozen wood frogs and proposed a novel freeze-responsive function of MEF2 proteins (Aguilar, Hadj-Moussa & Storey, 2017). MEF2 activity has been shown to be enhanced in response to extracellular signals which induce intracellular signaling cascades leading to MEF2 phosphorylation. PTMs, especially phosphorylation, are a major form of regulation used to coordinate metabolic depression and reorganization as they are readily reversible, energy-inexpensive, and easily inducible (Storey, 2015; Storey & Storey, 2004b). MEF2 proteins are phosphorylated by the MAP kinases ERK5 and p38 in response to osmotic and oxidative stress, respectively, suggesting a role of MAPKs in the anoxia-induced MEF2 response seen in this study (Potthoff & Olson, 2007; Son et al., 2011; Wang et al., 2006). In fact, past studies have characterized MAPKs as key players in stress survival, as seen during torpor in the gray mouse lemur, hibernation in the little brown bat, and hibernation in arctic ground squirrels (Biggar et al., 2015; Eddy & Storey, 2007; Zhu et al., 2005).

MEF2 proteins were overall found to be non-responsive to dehydration. Analysis of relative *mef2a* and *mef2c* mRNA abundance during 40% dehydration showed that both *mef2a* and *mef2c* remained constant (Fig. 1). Likewise, total MEF2A protein levels remained constant during 40% dehydration and rehydration, whereas MEF2C was unchanged during dehydration but downregulated during rehydration (Fig. 2). This result suggests that MEF2C may not be required for transitions between dehydration and rehydration state, consequently the wood frog may be downregulating the translation of MEF2C to conserve energy. Since post-translational modifications of MEF2 proteins play an important role in MEF2 activity, phosphorylation of MEF2A/C was examined. Phospho-MEF2A^Thr312^ and phospho-MEF2C^Thr300^ levels were found to remain constant during 40% dehydration and rehydration in comparison to control conditions, indicating that the total pool of active MEF2 protein is unaffected by dehydration/rehydration conditions (Fig. 2). These results were further supported by analysing the cytoplasmic and nuclear distribution of total and phosphorylated MEF2 proteins. Cytoplasmic and nuclear MEF2A/C and phospho-MEF2A/C levels were found to remain constant during 40% dehydration in comparison to control conditions (Figs. 4 and 5). Since MEF2 proteins are translocated from the cytoplasm to the nucleus where they are phosphorylated, these results indicate that MEF2 translation and activation is unchanged in response to dehydration (Black & Olson, 1998). Overall, these results suggest that MEF2 proteins do not respond to dehydration stress in wood frog skeletal muscle and that MEF2 proteins are not essential for transitions between dehydration and rehydration.

This study demonstrated a novel anoxia-mediated MEF2 response in wood frog skeletal muscles through translational regulation, nuclear localization, activating MEF2 phosphorylations, and transcriptional control of a select downstream target. Transcript analysis of *mef2a* and *mef2c* during 24 h anoxia showed that mRNA levels remained constant, relative to control conditions, suggesting that MEF2 proteins are not regulated at the transcriptional level during 24 h anoxia (Fig. 1). To further analyse MEF2 regulation, translational and post-translational regulation of MEF2A/C was examined by measuring total and phosphorylated MEF2A/C protein abundance at 4 h and 24 h anoxia. It was found that total and phospho-MEF2A^Thr312^ protein abundance remained constant during 4 h and 24 h anoxia (Fig. 3). In contrast, total MEF2C protein abundance remained constant during 4 h anoxia and was downregulated during 24 h anoxia, whereas phospho-MEF2C^Thr300^ increased during 4 h anoxia and remained constant during 24 h anoxia (Fig. 3). The anoxia-responsive increase in phosphorylated MEF2C protein abundance during 4 h anoxia indicates an overall increase in active MEF2C pools, suggesting that MEF2C proteins are required in the early anoxic transition phase, a phase that is also present in freezing. Changes in molecular processes in the wood frog occur within minutes of initial ice formation on the skin surface, and by 24 h maximum ice formation and anoxia stress have been reached (Storey, 2004; Storey & Storey, 1996; Storey & Storey, 2017). As such, the wood frog may require MEF2 activity in early anoxia stress to induce expression of downstream targets required for freeze tolerance throughout the winter. The decrease in total MEF2C expression during 24 h anoxia may be due to post-transcriptional mechanisms such as mRNA instability and decay, microRNA-directed mRNA cleavage, or accumulation of mRNA in cytoplasmic P-bodies and stress granules (Storey, 2015; Tessier & Storey, 2014). Past research has shown that the wood frog uses microRNA as a method of rapid, reversible gene silencing during freezing (Bansal, Luu & Storey, 2016; Biggar, Dubuc & Storey, 2009). In addition, a study on hibernating South American marsupials showed an upregulation of skeletal muscle microRNAs implicated in MEF2 regulation (Hadj-Moussa et al., 2016). As such, similar microRNAs may also be involved in MEF2 regulation in the wood frog skeletal muscle during anoxia. Studies have also suggested that the wood frog sequesters mRNA transcripts in P-bodies and stress granules to reduce translation and conserve energy during hypometabolism, which also allows for rapid translation of genes during thawing, and may be responsible for the decrease in MEF2C expression seen in this experiment, however further studies are required to elucidate molecular mechanisms responsible for this finding. (Al-Attar, Zhang & Storey, 2017; Aguilar, Hadj-Moussa & Storey, 2016; Bansal, Luu & Storey, 2016). Although analysis of total MEF2 levels only indicated an increase in MEF2C activity at 4 h anoxia, upon examination of the cytoplasmic and nuclear distribution of MEF2A/C it was found that both MEF2A and MEF2C are implicated in anoxia survival following 24 h anoxia stress. It was shown that during 24 h anoxia, the inactive cytoplasmic form of MEF2A was upregulated, and that active nuclear phospho-MEF2A^Thr312^ and phospho-MEF2C^Thr300^ levels also increased (Figs. 4 and 5). These results indicate an increase in MEF2A translation, and MEF2A/C nuclear localization and phosphorylation to generate a larger pool of active MEF2 protein in the nucleus required for anoxia survival. Inconsistencies between total and cytoplasmic MEF2A/C levels suggest a localized upregulation of MEF2 proteins in the cytoplasm. Overall, this study suggests that the MEF2 freeze response seen by Aguilar, Hadj-Moussa & Storey (2017) may have been induced by both freezing and the component stress of anoxia.

This study found an increase in MEF2A translational and post-translational regulation and MEF2C post-translational regulation in the wood frog skeletal muscle during anoxia, whereas MEF2A/C protein abundance remained unchanged during dehydration. To further analyse the implication of MEF2 proteins in dehydration and anoxia stress, transcript levels of four select MEF2 downstream targets required for muscle functioning and pro-survival processes were analysed during 40% dehydration and 24 h anoxia. *Glut4* transcript levels remained constant during 40% dehydration, whereas transcript levels were increased during 24 h anoxia (Fig. 6), which corresponded to the MEF2 expression observed in this study. Glucose is the main wood frog cryoprotectant that is used to reduce ice formation and excessive cellular dehydration (Amaral, Lee & Costanzo, 2013; Storey & Storey, 1984). Within minutes of initial ice formation on the skin surface, the wood frog will activate liver glycogenolysis to increase plasma glucose levels to 200–300 mM, compared to ∼5 mM in control frogs, allowing uptake of glucose into organs. The rate of glucose uptake is largely dependent on the abundance of glucose transporters in the plasma membrane (Costanzo & Lee Jr, 1993; Storey, 1987a; Storey & Storey, 2004b; Storey & Storey, 2004c). The GLUT4 transporter is a highly regulated transporter which is under the transcriptional control of the MEF2 factors, as well as Rac1, the GLUT4 Enhancer Factor (GEF), and Myogenic Differentiation Factor D (MyoD) (Chiu et al., 2011; Knight et al., 2003; Zorzano, Palacín & Gumà, 2005). The importance of glucose and glucose transporters for freeze tolerance has been shown in numerous studies (Costanzo & Lee Jr, 1993; Holden & Storey, 2000; King, Rosholt & Storey, 1995; Storey, 1987a; Storey, 2004). Results from the present study further support past research and suggest that the increase in GLUT4 and glucose uptake may stem from the component stress of anoxia. Likewise, a study on wood frog liver revealed an increase in GLUT2 expression under hypoxic conditions, indicating a more global response of GLUT proteins to anoxia stress (Rosendale, Lee & Costanzo, 2014). An increase in GLUT expression in response to hypoxic/anoxic conditions has also been shown in various animals including GLUT2 in sea bass liver, and GLUT1 in rat cardiac muscle (Sivitz et al., 1992; Terova et al., 2009).

Calreticulin is also under the transcriptional regulation of MEF2 proteins. Calreticulin is an endoplasmic reticulum lumen-residing protein with two main functions: maintaining Ca^2 +^ homeostasis and maintaining protein quality control through protein chaperoning (Lu, Weng & Lee, 2015; Michalak et al., 1999). During 40% dehydration, *calr* transcript levels remained constant which coincides with MEF2 expression observed during dehydration (Fig. 6). *Calr* levels were found to remain constant during 24 h which was inconsistent with MEF2 expression, suggesting that transcriptional regulation of calreticulin by other transcription factors may be predominant in wood frog skeletal muscle during anoxia. Indeed, calreticulin has been shown to be regulated by the transcription factors GATA6, NKx2.5, PPAR factors, COUP-TF1, and Evi-1, and whether these proteins regulate calreticulin in the wood frog skeletal muscle requires further study (Lu, Weng & Lee, 2015). The results obtained in this study may also suggest that calreticulin is freeze-responsive rather than dehydration or anoxia-responsive, as seen by Aguilar, Hadj-Moussa & Storey (2017). As such, other chaperone proteins may be upregulated in wood frog skeletal muscle in response to dehydration and anoxia stress. Chaperone proteins are essential to maintain protein folding, trafficking, and assembly under stress (Storey & Storey, 2017). Heat shock proteins (HSP) are the best-known stress-induced chaperones upregulated in response to freezing and oxygen limitations (Storey & Storey, 2017). Studies have shown increased levels of HSPs in winter frogs, painted and soft-shelled turtles, and gray mouse lemurs under stress (Chang, Knowlton & Wasser, 2000; Kiss et al., 2011; Wu et al., 2015). HSPs have also been found to be upregulated in the muscle of red-eared slider turtles and painted turtles under anoxic conditions, suggesting a dominant role for HSPs during anoxia stress rather than calreticulin (Krivoruchko & Storey, 2010). Differential expression of HSP in *Xenopus laevis* skeletal muscle during dehydration stress also suggests that HSPs play a dominant role in dehydration stress response (Luu et al., 2017). Further experimentation is required to determine whether the wood frog favours HSPs to survive dehydration and anoxia stress.

MEF2 proteins also regulate the transcriptional expression of muscle and brain creatine kinase isozymes (Horlick et al., 1990; Satyaraj & Storb, 1998). Muscle and brain creatine kinase are both expressed ubiquitously, with their name indicating the tissue in which they are predominantly expressed (Horlick et al., 1990). The creatine kinase isozymes have key functions in the phosphocreatine circuit which maintains cellular energy homeostasis by controlling phosphagen pools. Creatine kinase catalyses the transfer of a high-energy phosphate from ATP to creatine to produce phosphocreatine which serves as an energy reserve. CK is abundant in energy-demanding tissues such as the muscle which requires rapid ATP-turnover (Baird et al., 2012; Pfefferle et al., 2011; Wallimann et al., 1992). Prior research has shown that CK activity is upregulated in the frozen wood frog skeletal muscle which is important in maintaining muscle energetics during freezing and thawing (Aguilar, Hadj-Moussa & Storey, 2017; Dieni & Storey, 2009). The present study found that *ckm* and *ckb* transcript levels remained constant during 40% dehydration and 24 h anoxia, suggesting that creatine kinase is freezing-responsive rather than dehydration or anoxia-responsive (Fig. 6). As such, the wood frog may employ other biochemical mechanisms to maintain energy metabolism in response to anoxia and dehydration stress. AMP deaminase (AMPD) and adenylate kinase play a critical role in stabilizing ATP, ADP, and AMP pools when ATP consumption exceeds ATP production (Storey & Storey, 2017). AMPD has been shown to be upregulated in skeletal muscle of red-eared slider turtles under anoxic conditions, (Zhou, 2006). Similarly, AMPD activity has been shown to increase in the wood frog skeletal muscle in response to freezing, however whether this response is trigger by the component stresses of anoxia or dehydration has yet to be elucidated (Dieni & Storey, 2008).

In summary, this study identified a novel MEF2 response to anoxia stress in the skeletal muscle of the wood frog. This response was shown to be mediated through translational regulation, nuclear localization, and phosphorylation of MEF2 proteins during anoxia, inducing an upregulation of *glut4* which is critical for the distribution of glucose as a cryoprotectant in the muscle. In contrast, expression of MEF2 proteins and their downstream targets were found to remain unchanged during dehydration stress. The upregulation of MEF2 activity during anoxia stress suggests that the previously characterized freeze-responsive upregulation of wood frog MEF2s may in part stem from their cellular responses to surviving prolonged anoxia rather than dehydration. Examining the molecular mechanisms employed by the wood frog to survive freezing, anoxia, and dehydration helps us to dissect out stress responses and to better understand their molecular underpinnings.

##  Supplemental Information

10.7717/peerj.4014/supp-1 Data S1 Immunoblots (raw data)

10.7717/peerj.4014/supp-2 Data S2 Normalized data for Figs. 1–5

## Additional Information and Declarations

### Data Availability

The following information was supplied regarding data availability: The raw data has been provided as Supplemental Files.
