# Supplementary material for: The role of MEF2 transcription factors in dehydration and anoxia survival in Rana sylvatica skeletal muscle
Source: PeerJ. 2017 Nov 9;5:e4014. doi: 10.7717/peerj.4014 (PMC5682099; doi:10.7717/peerj.4014)
Supplement: Data S1 [file peerj-05-4014-s001.pptx]

## Slide 1
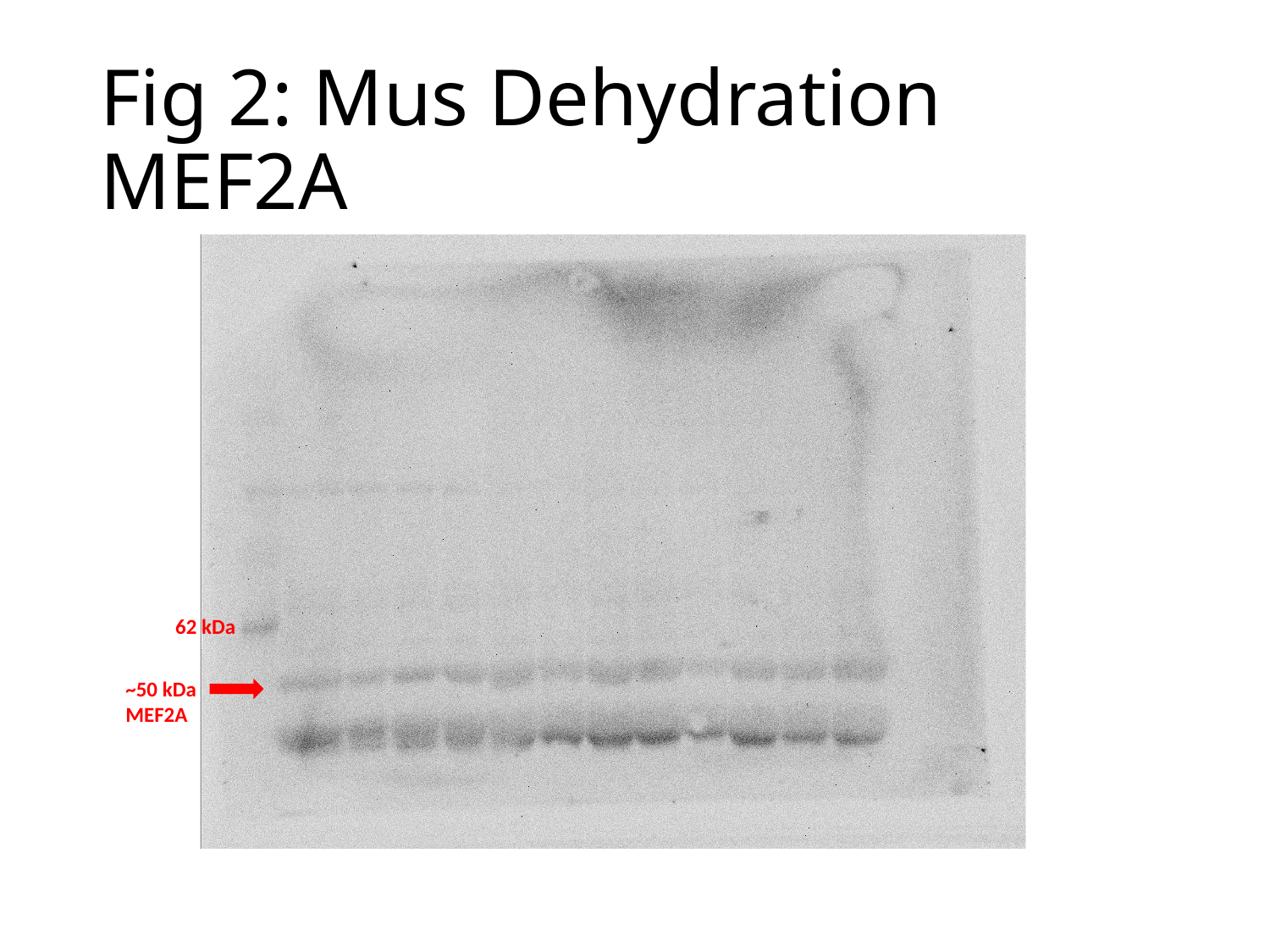

# Fig 2: Mus Dehydration MEF2A
62 kDa
~50 kDa
MEF2A

## Slide 2
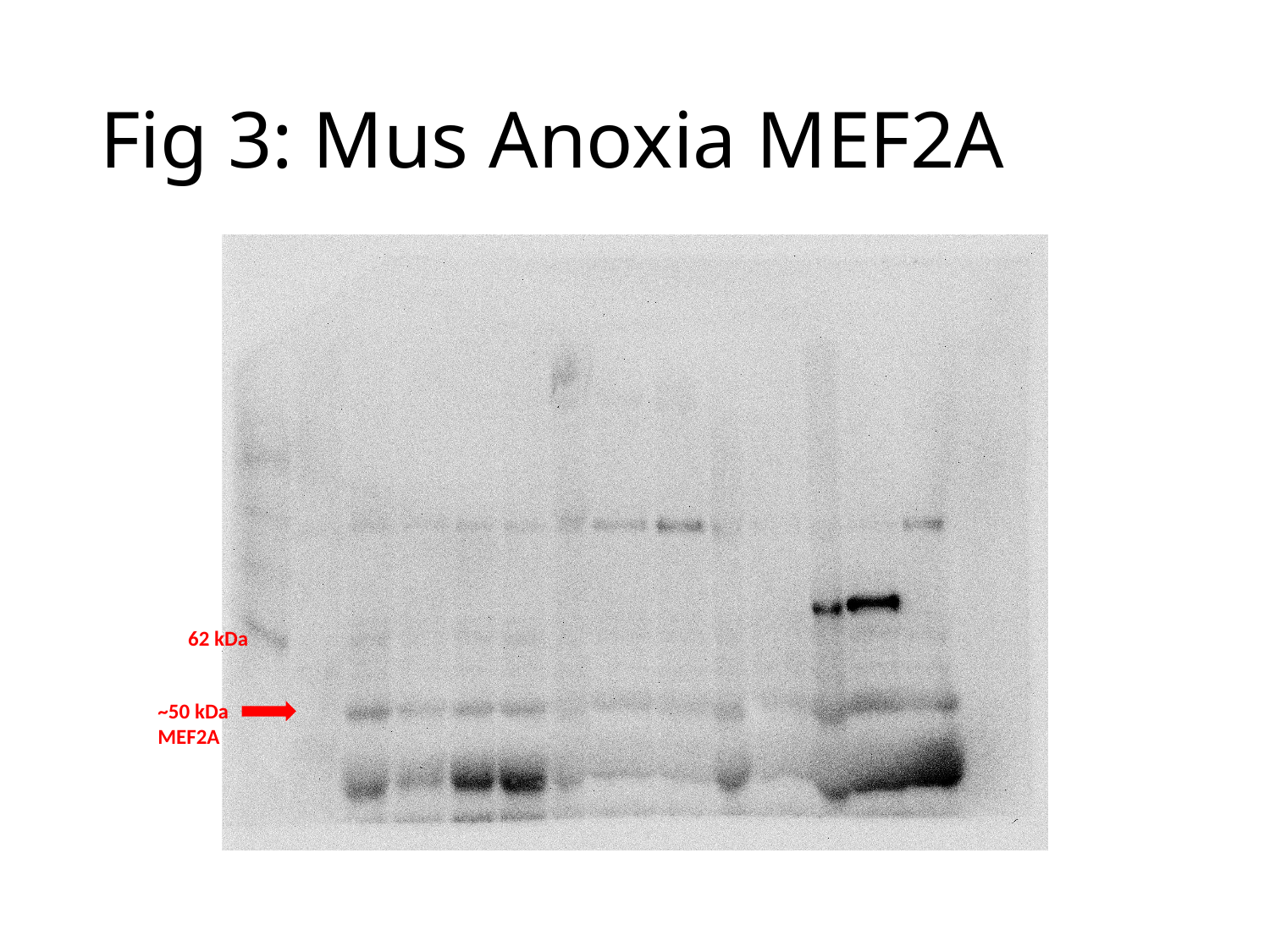

# Fig 3: Mus Anoxia MEF2A
62 kDa
~50 kDa
MEF2A

## Slide 3
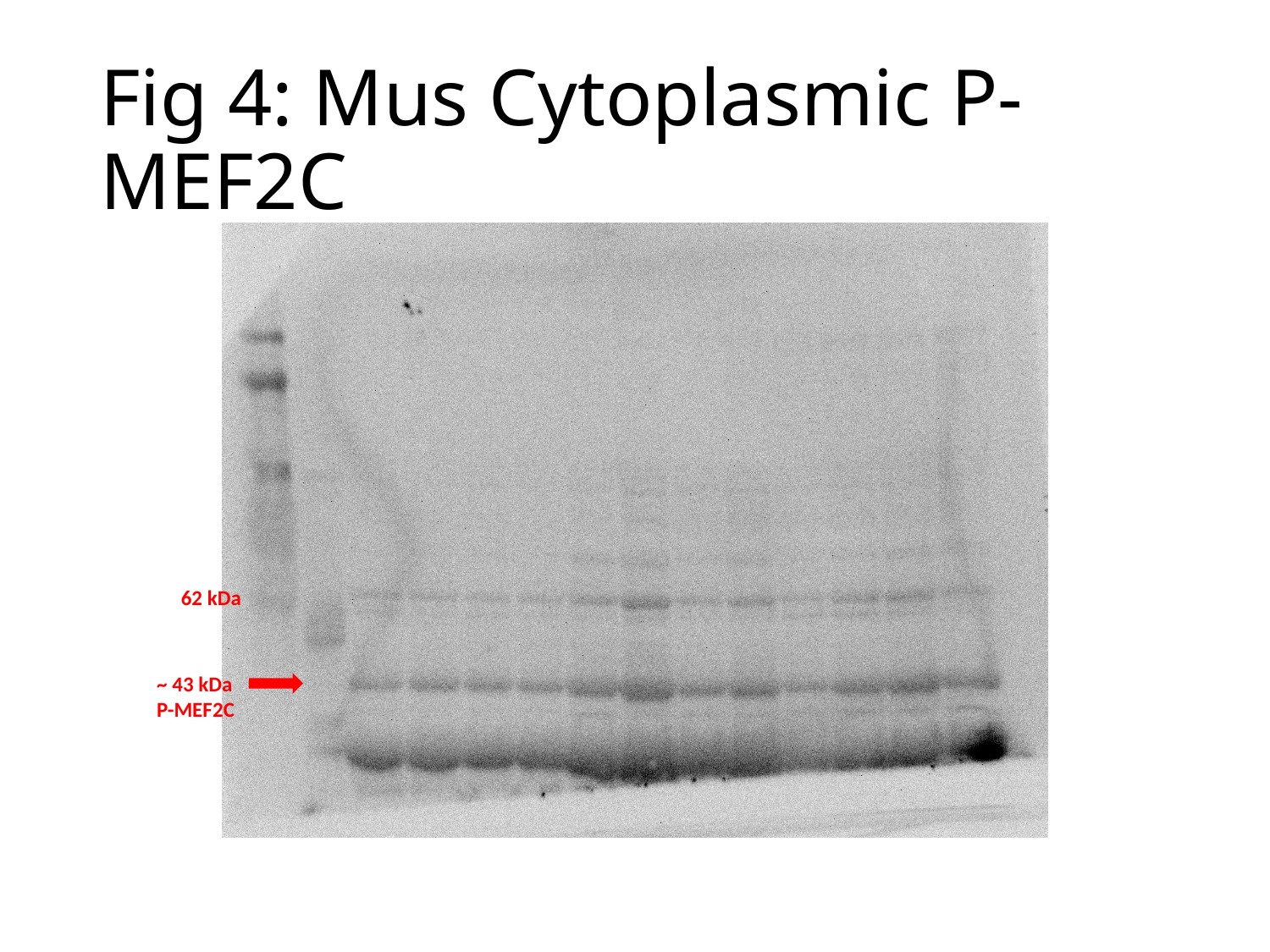

# Fig 4: Mus Cytoplasmic P-MEF2C
62 kDa
~ 43 kDa
P-MEF2C

## Slide 4
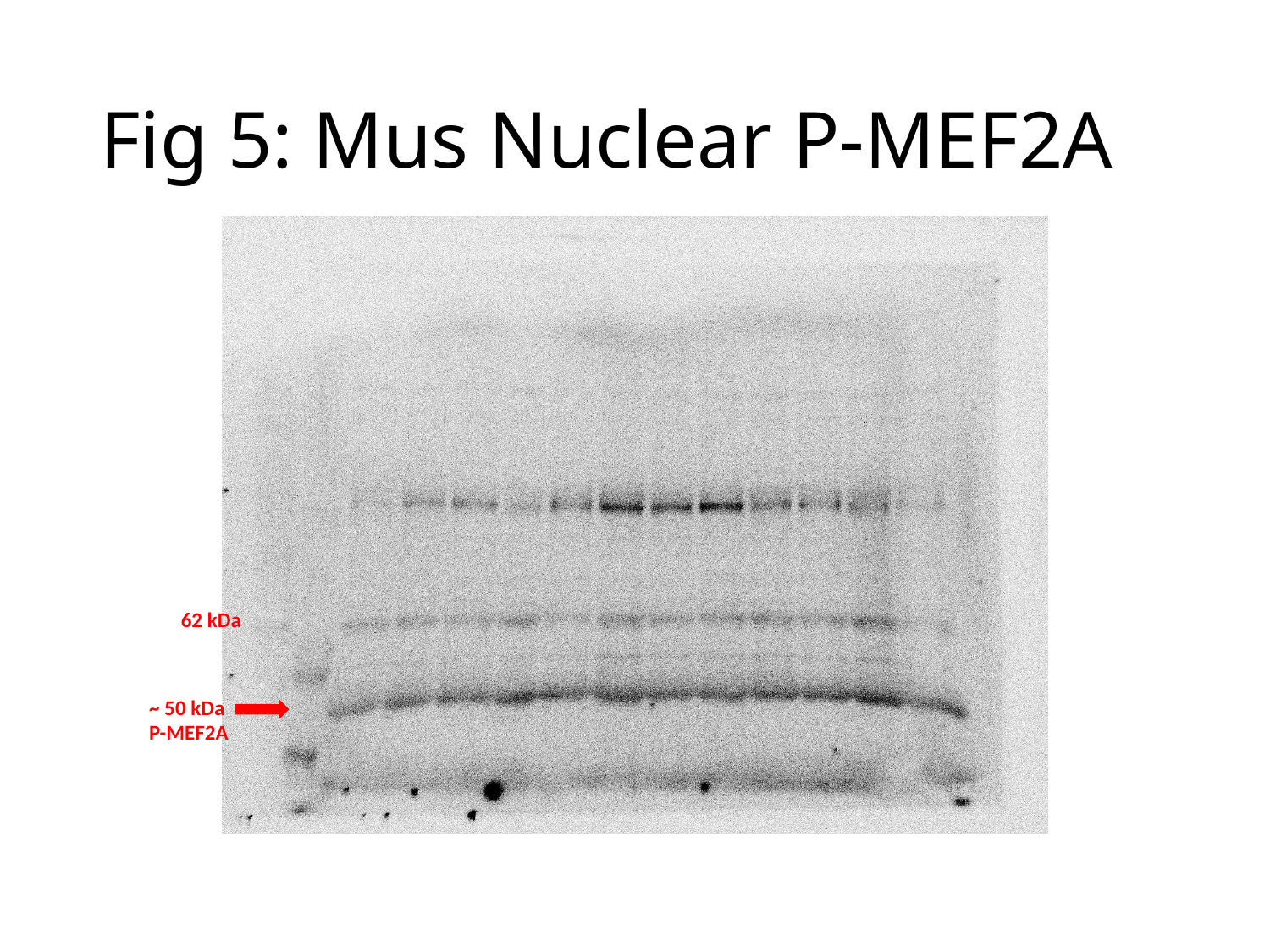

# Fig 5: Mus Nuclear P-MEF2A
62 kDa
~ 50 kDa
P-MEF2A
